# Temporal Dynamics of Pain: Examining the Relationship Between Intramuscular Injection Duration and Pain Perception: A Crossover Randomized Controlled Trial

**DOI:** 10.7759/cureus.70969

**Published:** 2024-10-06

**Authors:** Yusuf Acarlioglu, Leman Senturan, Saleh Salimi

**Affiliations:** 1 Civil Defense and Mobilization, Istanbul Provincial Health Directorate, Istanbul, TUR; 2 Nursing, Faculty of Health Sciences, Biruni University, Istanbul, TUR

**Keywords:** injection duration, intramuscular injection, nursing, nursing administration, pain perception

## Abstract

Background

Effective pain management is crucial for optimizing the patient experience and satisfaction with care. Many factors affect the intensity of injection pain, and one of these factors is the duration of the injection. This study aimed to evaluate the impact of intramuscular injection duration on perceived pain intensity in adults.

Methods

Fifty participants received injections according to a predetermined research protocol. Each participant served as their own control group. The intervention group was administered the drug over 40 seconds. The control group was given the same drug on the other side dorsogluteal region the following day, according to the unit's routine, in 10 seconds. Pain intensity was measured using the Wong-Baker Faces Pain Rating Scale and the Numerical Rating Scale. Statistical analysis was performed using SPSS (IBM Corp. Released 2017. IBM SPSS Statistics for Windows, Version 25.0. Armonk, NY: IBM Corp), including Wilcoxon and correlation analyses, with a significance level set at p < 0.05.

Results

The Wilcoxon test revealed a significant decrease in pain scores for the intervention group (p < 0.05), indicating that longer injection durations were associated with lower pain intensity. Significant correlations were found between hospital phobia and the use of pain relief measures, with pain perception (p < 0.05).

Conclusion

This research establishes a correlation between extended durations of intramuscular injections and pain intensity. These findings have implications for pain management during medical procedures and can contribute to optimizing patient comfort.

## Introduction

Intramuscular injection (IM) is a common method of drug administration in healthcare settings [1]. The World Health Organization (WHO) reports that 16 billion injections are administered worldwide annually, and 90% are administered intramuscularly. Although IM injections are generally safe and effective, pain at the injection site is a common side effect, causing discomfort and anxiety, which may negatively affect patient compliance and satisfaction with treatment and care [2].

Pain adversely affects a person's social life, physical and mental health, and overall quality of life. Due to its significant impact on patient outcomes, pain is now considered the fifth vital sign, alongside temperature, pulse, respiration, and blood pressure [3]. Although the impact of acute pain is less explored in the literature, its significance in nursing and patient satisfaction cannot be overlooked. Given the ongoing debate about the optimal duration of intramuscular injections to minimize or alleviate pain, this innovative randomized controlled trial design may provide valuable insights. Research on factors influencing perceived IM pain classifies it into three subdomains: drug formulation, patient factors, and injection-process-related factors. Drug formulation, particularly the pH, osmolality, excipients, and administered volume are significant determinants of perceived pain intensity [4]. Drug formulations should target a pH as close as possible to the physiological pH to reduce pain and tissue damage [5]. To prevent pain, injectable products should be formulated as isotonic solutions (osmolality around 300 mOsm/kg), ideally not exceeding 600 mOsm/kg [6]. Excipients, chemical compounds added to drug formulations to improve efficacy and stability, are essential, although concerns about potential allergic reactions persist. Despite these concerns, their use is often unavoidable.

Injection process-related factors also influence perceived pain intensity. St. Clair-Jones et al. discussed the impact of injection frequency, injection angle/technique, injection site, the temperature of the biologic solution, and hypersensitivity [4]. Jamalinik et al. highlighted the benefits of applying ice or lidocaine spray to reduce IM injection pain [7]. Jancy reported that using a cold needle (0-2°C) effectively reduces pain perception during IM injections [8]. Zeyrek et al. recommended using smaller needle sizes, the Z-technique, administering injections in the ventrogluteal (VG) site, and applying manual pressure [9]. Patient-related factors are the third class of factors influencing perceived pain intensity. Fear of injection, poor sleep before injection, female gender, low body weight, fibromyalgia, depression, and severe rheumatoid arthritis have been independently associated with a significantly increased likelihood of experiencing greater injection pain [4,10,11].

This study focuses on the duration of IM injection, a potential factor affecting perceived pain intensity. In a systematic review on this subject, several studies have investigated the impact of injection duration on pain intensity, but the results have been inconsistent [1]. Some argue that slow injections allow muscle fibers to stretch, facilitating better retention of the drug and minimizing the risk of leakage along the needle track [9,12,13]. One study, however, found no significant difference in pain intensity between fast and slow injection speeds [14]. Given the prevalence of intramuscular injections and their impact on patient satisfaction, along with the inconsistent findings in this topic, further research is needed to identify the optimal injection duration to enhance patient comfort and compliance [2]. Therefore, this study aims to investigate the impact of IM injection duration on the pain intensity perceived by patients.

## Materials and methods

This study was a single-group crossover randomized controlled trial. In this type of clinical trial design, all participants receive multiple interventions at different times, allowing each participant to serve as their own control. This design enables the comparison of different interventions within the same individual.

The study aimed to evaluate the impact of intramuscular (IM) injection duration on perceived pain intensity in adults. It was conducted in the emergency unit of an educational hospital in Istanbul.

Participants

The study enrolled 50 participants who presented to the emergency unit of an educational hospital for IM injections ordered by physicians. The sample size was calculated using G*Power software, based on a dependent t-test with a Cohen's d effect size of 0.4, an alpha of 0.05, and a power of 0.80. The required sample size per group was 48 participants, so the study aimed to enroll a total of 50 participants. Inclusion criteria included being aged between 18 and older, having a normal body mass index (BMI), having a prescription for at least two doses of IM clindamycin (Klindan 600 mg/4 ml, manufactured by Bilim Drug Company), speaking fluent Turkish, and having no damage in the dorsogluteal region (DGR) tissue of both limbs (including pain, hematoma, necrosis, scar, incision, or infection symptoms on the skin). Participants were also required to have no history of injection in the past two weeks, surgery, or botox in the injection area, and to have signed informed consent after an oral explanation of the study.

Exclusion criteria included reporting any pain at the injection site, experiencing complications from the first injection, refusing to continue participating in the study for the second injection, or taking painkillers, including nonsteroidal anti-inflammatory drugs and acetaminophen, within 12 hours before the injection.

**Figure 1:**
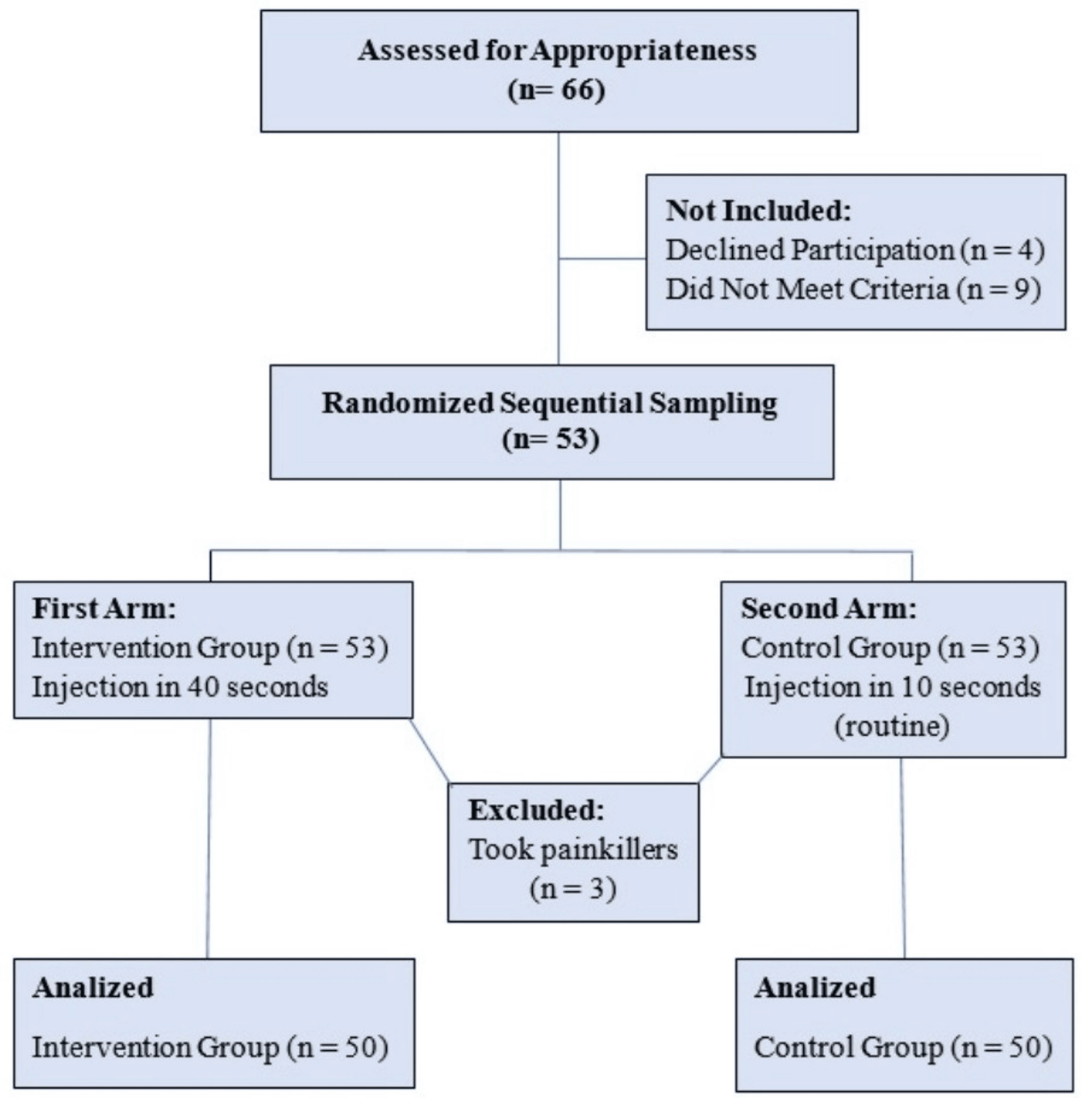
Participant Flow Diagram

A sequential sampling approach was used for participant recruitment. All participants who met the inclusion criteria were selected as samples, and the defined sample size was reached within three months. Participants were randomly allocated to the intervention and control groups. Allocation was done using a computer-generated random number table with a cutoff point of 0.5. The randomization of injection sites (right or left DGR) was also carried out using computer-generated random number tables, ensuring random assignment for each site. Each participant acted as their own control, resulting in 50 participants in each group (Figure 1). Each client received at least two clindamycin IM injections 24 hours apart, and if they received more, only the first two were evaluated. The demographic form was completed by one researcher, and other forms were filled out twice-immediately after the first and second injections.

Procedures and data collection tools

Pain was measured immediately after the injections using two scales: the Wong-Baker Faces Pain Rating Scale (WBFS) and the Numerical Rating Scale (NRS). Both were administered by one of the researchers. The WBFS assigns a numerical rating to each face on the scale, ranging from 0 (no hurt) to 10 (hurts worst) [15]. In the Turkish version used in this study, the scale ranged from 0 to 5, with its validity and reliability confirmed by Tufekci and Behice [16]. The NRS is a global scale that uses numbers from 0 to 10, with 0 indicating no pain and 10 indicating unbearable pain [17]. Permission was granted by the authors and translators of both scales. The demographic form collected background data before the first injection, including age, gender, weight, height, chronic conditions, surgery history at the injection site, IM injection history, and hospital phobia.

The study consisted of two arms - the intervention group, in which the drug was administered randomly to one of the participant's DGR regions over 40 seconds, and the control group, where the same drug was administered to the other DGR region according to the unit's routine over 10 seconds on the following day. To minimize the impact of various factors on perceived pain, we used the same drug formulation (Klindan 600 mg/4 ml, manufactured by Bilim Drug Company) was administered by a single trained nurse to all participants in both arms. The nurse, who holds a B.S. degree and has six years of experience working in the emergency department, received specific training for the injections.

During the procedure, the participant was placed in the prone position, and the DGR region was selected for the injections. A standard 5cc injection needle with a 21-gauge size, manufactured by one company, was used for all injections. After reconstituting and drawing the drug into the syringe, the nurse changed the needle and used the solvent at room temperature. The use of ice, heat, or cooling of the needle, as well as the addition of lidocaine to the drug, was prohibited.

To further minimize patient-related factors, participants were required to have a normal BMI, at least six hours of sleep the night before, and no history of fibromyalgia, depression, or rheumatoid arthritis. This was a single-blinded randomized controlled trial, meaning the participants were unaware of which injection was for the control or intervention group.

Ethical consideration

The study was approved by the Ethics Committee of Biruni University (2019/25-57) and the Istanbul Governorship Provincial Health Directorate (16867222-604.01.01) for its conduct at the Istanbul Education and Research Hospital. The study was registered at the National Library of Medicine (ID: NCT05822336). Participants received an oral explanation about the research and signed informed consent forms.

Conflict of interest disclosures

The authors declare that they have no conflicts of interest. The study was registered with the National Library of Medicine (ID: NCT05822336).

Statistical analysis

Descriptive statistics are presented as mean ± standard deviation for numerical variables and as numbers and percentages for categorical variables. Statistical analysis was performed using SPSS (IBM Corp. Released 2017. IBM SPSS Statistics for Windows, Version 25.0. Armonk, NY: IBM Corp), including the Kolmogorov-Smirnov test, Mann-Whitney U test, Wilcoxon test, and Spearman correlation analysis. The significance level was set at p < 0.05. The questionnaire was anonymous and coded by the responsible researcher.

## Results

The characteristics of the participants are displayed in Table 1. The mean age was 31.7 ± 10.9 years. Of the participants, 56% (n=28) were male, 38% (n=19) had completed primary school, 14% (n=7) had a chronic condition, 36% (n=18) had a history of surgery, and 18% (n=9) reported hospital phobia. Among the participants with chronic conditions (n=7, 14%), two had asthma, four had cardiovascular diseases, and one had goiter. The majority of the participants had prior experience with IM injections, did not believe that IM injection pain was psychological, and reported no fear of injections or use of pain relief measures for IM injection pain. This variable refers to whether participants perceive the pain associated with IM injections as primarily psychological rather than physical. This distinction is important for understanding whether psychological factors influence pain perception and reporting.

**Table 1:** Characteristics of the Participants (N=50)

Characteristics		n (%)
Gender	Woman	22 (44)
	Men	28 (56)
Educational status	Primary school	19 (38)
	High School	24 (48)
	University Degree	7 (14)
Employment status	Unemployed	20 (40)
	Employed	30 (60)
Chronic condition	No	43 (86)
	Yes	7 (14)
History of surgery	No	32 (64)
	Yes	18 (36)
Hospital Fobia	No	41 (82)
	Yes	9 (18)
History of previous IM injection	No	5 (10)
	Yes	45 (90)
Considering IM pain as a kind of psychological pain	No	33 (66)
	Yes	17 (34)
Applying measures for IM pain	No	43 (86)
	Yes	7 (14)
Fear of injection	None	30 (60)
	Little	15 (30)
	Moderate	3 (6)
	A lot	2 (4)
	Mean±SD	Min-Max
Age (years)	31.7 ± 10.9	18 - 55
Weight (kg)	71.5 ± 14.5	40 - 110
Height (cm)	170 ± 9.6	154 - 193
Body Mass Index (kg/m^2^)	24.6 ± 4.3	16.6 - 40.6

Table 2 shows the differences in pain scores between the control and intervention groups, revealing a statistically significant reduction in pain scores in the intervention group. The results indicate a pattern of pain reduction on both the Wong-Baker Faces Pain Rating Scale (WBFS) and the Numerical Rating Scale (NRS), with prolonged injection duration associated with decreased pain intensity. There was no statistically significant correlation between age, BMI, and perceived IM pain severity.

**Table 2:** Perceived Pain Intensity Based on Age and BMI in the Control and Intervention Groups WBFS = Wong–Baker Faces Pain Rating Scale; NRS = Numerical Rating Scale. *Wilcoxon test for differences in pain intensity scores; **Pearson Correlation Analysis for correlations with Age and BMI. p-values from the Wilcoxon test assess differences in pain intensity scores, with significance at p < 0.001. z-scores indicate the magnitude of these differences. r-values represent the strength and direction of correlations between pain intensity and Age/BMI, with significance at p < 0.05.

Scale and Groups	Pain Intensity Score					Correlation			
						Age		BMI	
	Min	Max	Mean (SD)	p	z*	p	r**	p	r**
WBFS- Control Group	0	5.0	2.64 (1.27)	0.000	-5.194	-0.222	0.121	-0.217	0.131
WBFS- Intervention Group	0	5.0	1.60 (1.06)			-0.009	0.950	-0.256	0.072
NRS- Control Group	0	10.0	4.81 (2.60)	0.000	-4.924	-0.223	0.119	-0.187	0.193
NRS- Intervention Group	0	9.8	2.64 (2.23)			-0.008	0.957	-0.213	0.138

Data analyses also found no significant correlation between chronic condition, surgery history, IM injection history, attitudes toward pain, and perceived pain severity (Table 3). However, in the intervention group, a statistically significant correlation was found between hospital phobia and using pain relief measures only on the NRS scale. Participants who reported hospital phobia and used pain relief methods experienced higher perceived pain scores.

**Table 3:** Comparison of Mean Pain Intensity Between Groups Across Various Variables WBFS: Wong–Baker Faces Pain Rating Scale; NRS: Numerical Rating Scale; *Mann-Whitney U test. p values indicate statistical significance at p < 0.05. z scores reflect the magnitude of differences between groups, with larger absolute values denoting greater differences.

Variable		n	WBFC- Control			WBFC- Intervention			NRS- Control			NRS- Intervention		
			Mean (SD)	p	z*	Mean (SD)	p	z*	Mean (SD)	p	z*	Mean (SD)	p	z*
Gender	Men	28	2.46 (0 .99)	0.21	-1.12	1.39 (0.76)	0.16	-1.39	4.96 (2.65)	0.74	-0.32	2.57(2.37)	0.56	0.58
	Women	22	2.86 (1.55)			1.86 (1.24)			4.73 (2.62)			2.82 (2.10)		
Chronic diseases	No	43	2.70 (1.24)	0.52	-0.65	1.53 (1.05)	0.19	-1.32	4.91 (2.52)	0.68	-0.40	2.84 (2.27)	0.24	-1.16
	Yes	7	2.29 (1.49)			2.00 (1.15)			4.57 (3.35)			1.71 (1.88)		
History of surgery	No	32	2.84 (1.22)	0.20	-1.27	1.78 (1.09)	0.19	-1.30	5.34 (2.48)	0.07	-1.77	3.00 (2.38)	0.21	-1.23
	Yes	18	2.28 (1.32)			1.28 (0.96)			4.00 (2.70)			2.11 (1.90)		
Hospital phobia	No	41	2.68 (1.29)	0.45	-0.74	1.61 (1.09)	0.92	-0.09	4.54 (2.65)	0.07	-1.80	2.20 (1.86)	0.004	-2.87
	Yes	9	2.44 (1.23)			1.56 (1.01)			6.33 (1.93)			4.89 (2.61)		
Pain relief measures	No	43	2.63 (1.32)	0.25	-1.12	1.58 (1.07)	0.24	-1.15	4.86 (2.57)	0.24	-1.15	2.49 (2.22)	0.01	-2.46
	Yes	7	2.71 (0.95)			1.71 (1.11)			4.86 (3.07)			3.86 (2.11)		
History of injection	No	5	3.20 (1.30)	0.80	-0.24	2.00 ( 0.70)	0.72	-0.35	4.40 (3.64)	0.11	11.59	3.60 (4.03)	0.80	-1.74
	Yes	45	2.58 (1.27)			1.56 (1.09)			4.91 (2.53)			2.58 (2.00)		
Considering IM pain as psychological pain	No	33	2.52 (1.39)	0.81	-0.23	1.36 (0.99)	0.51	-0.65	5.24 (2.66)	0.64	-0.46	2.88 (2.26)	0.64	-0.46
	Yes	17	2.88 (0.99)			2.06 (1.08)			4.12 (2.42)			2.29 (2.22)		

## Discussion

The primary objective of this study was to investigate the relationship between IM injection duration and the severity of perceived pain. Our findings substantiate the initial hypothesis, showing a clear association between prolonged injection duration and a reduction in the intensity of pain experienced during IM injections. As shown in Table 2, the Wilcoxon test revealed a statistically significant reduction in pain scores for the intervention group (p < 0.05). This result supports the idea that longer injection durations are associated with lower pain levels, corroborating previous studies that identified similar correlations between these variables [9,12,13]. The convergence of findings across multiple studies suggests that the practice of prolonging IM injection duration can consistently reduce pain intensity across different patient populations. However, contrasting findings in the literature, which report no relationship between injection duration and pain intensity, merit consideration [14]. It is essential to acknowledge that our study employed rigorous methods to control confounding variables, as outlined in the methods section, strengthening the reliability of the results. Therefore, we advocate that extended injection duration is likely to alleviate pain intensity during IM injections.

When examining participant characteristics in relation to the severity of perceived pain, we found no significant correlations with variables such as the presence of chronic conditions, age, BMI, history of surgery, history of IM injections, or beliefs about the psychological nature of IM pain (Tables 2, 3). Nevertheless, participants who reported hospital phobia and employed post-injection pain relief methods exhibited significantly higher mean pain scores (Table 3). These findings align with existing literature, which underscores the psychological and emotional dimensions of pain perception [11].

Although some studies have suggested that pain perception may differ based on sex, potentially due to biological and hormonal differences [18,19], our study did not find a significant difference in pain intensity between male and female participants (p > 0.001; z = -1.12). This finding contradicts literature that generally indicates higher pain perception in females [20-22]. These discrepancies could be attributed to sample cultural characteristics, such as the relatively small sample size, as well as the complexity of pain perception mechanisms. Factors such as pain thresholds, coping strategies, and socio-cultural influences may also play a role in these variations.

The relationship between age and pain intensity has been inconsistently reported in the literature, with some studies suggesting a positive correlation, while others, reported no significant association [13,20, 22-24]. Our findings support the latter, indicating no significant correlation between age and pain intensity. The complex nature of pain perception, influenced by individual differences in sensory thresholds, pain expectations, and neurobiological factors, might explain these inconsistencies [19,25,26]. To draw definitive conclusions, future studies with larger sample sizes are warranted.

BMI and its correlation with pain intensity were also examined in this study. Consistent with findings from Suhrabi et al., Taghinejad et al., and Bilge et al. [20,24], we did not observe a significant relationship between BMI and pain scores (r = .131, p < 0.001). However, some studies have reported that higher BMI is associated with increased pain severity [13]. This discrepancy may be due to differences in methodology or patient populations. Similarly, we found no significant correlation between prior history of IM injections and pain intensity (U = 0.221, p < 0.05), contrasting with studies that reported higher pain severity in individuals with previous IM injection experience [25,27]. These conflicting findings underscore the need for further research to explore the moderating factors that may affect the relationship between prior injection history and pain perception.

The psychological dimension of pain was further explored by investigating the correlation between pain intensity and hospital phobia. Our results showed a statistically significant correlation (U = -2.08, p < 0.004), which suggests that individuals with heightened anxiety or fear related to the hospital environment may experience more intense pain during medical procedures [28,29]. This finding highlights the important role that psychological factors, such as anxiety and coping strategies, play in shaping pain experiences. Additionally, participants who used pain relief measures after injection reported higher pain levels (U = -2.46, p < 0.01), a result that is consistent with the broader literature on the subject [26]. This could suggest that individuals who are more proactive in seeking pain relief may also have higher pain sensitivity or anxiety related to injections.

Pain perception is a multifaceted phenomenon influenced by numerous individual and contextual factors. Our study contributes to the growing body of evidence supporting the link between prolonged injection durations and reductions in pain intensity. Furthermore, it reinforces the understanding that psychological factors, such as hospital phobia and pain relief behaviors, are closely intertwined with pain experiences. However, further research on this topic is essential to better understand the underlying mechanisms of these relationships, including potential sex differences and the roles of cultural, genetic, and psychosocial factors [18,25].

Limitations

The study's limitations include the potential for carryover effects, where the impact of the first injection may have influenced the second. The single-blinded design also introduced the possibility of bias due to the nurse's awareness of the study hypothesis. Changes in participants' mood on the days of injections may affect their pain perception, which was beyond our control. Furthermore, the cultural context may limit the generalizability of the results, as pain perception is influenced by cultural and individual beliefs.

## Conclusions

Our results demonstrate a statistically significant reduction in pain scores within the intervention group, indicating that a prolonged injection duration correlates with a measurable decrease in pain intensity. This relationship suggests that administering injections more slowly may help alleviate patient discomfort, as reflected in the observed trends in pain score patterns.

The findings carry important implications for optimizing pain management strategies in medical procedures. By incorporating prolonged injection duration into routine clinical practice, healthcare providers could enhance patient comfort, reduce anxiety, and potentially improve overall patient outcomes, such as drug compliance, treatment compliance, and other patient outcomes. These insights may lead to a decreased reliance on medication for managing IM injection pain, as noted by some participants.

Further research should investigate additional variables, such as individualized pain perception, cultural influences, and social modeling, to create a more holistic understanding of the factors that shape injection pain experiences. Such studies could provide deeper insights into personalized pain management strategies, leading to tailored interventions that are more effective across diverse patient populations.
